# TMT1A inhibits lung adenocarcinoma progression by suppressing M2 macrophage polarization

**DOI:** 10.1016/j.isci.2025.114332

**Published:** 2025-12-03

**Authors:** Junfan Pan, Qiongwen Wu, Yunan Zhao, Sixuan Wu, Liu He, Qihong Pan, Xiaohui Chen, Jing Zhang, Yiquan Xu

**Affiliations:** 1 Clinical Oncology School of Fujian Medical University, Fujian Cancer Hospital, Fuzhou 350014, China; 2 School of Basic Medical Sciences, Fujian Medical University, Fuzhou 350122, China; 3 Fuzhou University Affiliated Provincial Hospital, Fuzhou 350001, China; 4 Department of Oncology, The First Affiliated Hospital, Hengyang Medical School, University of South China, Hengyang, Hunan 421001, China; 5 Department of Thoracic Surgery, Clinical Oncology School of Fujian Medical University, Fujian Cancer Hospital, NHC Key Laboratory of Cancer Metabolism, Fuzhou 350014, China; 6 Interdisciplinary Institute of Medical Engineering of Fuzhou University, Fuzhou 350108, China; 7 Department of Thoracic Oncology, Clinical Oncology School of Fujian Medical University, Fujian Cancer Hospital, Fuzhou 350014, China

**Keywords:** immunology, cancer

## Abstract

The role of thiol methyltransferase 1A (TMT1A) in lung adenocarcinoma (LUAD) progression and the immune microenvironment remains unclear. Analysis of clinical samples and public databases revealed significantly lower TMT1A expression in tumorous LUAD samples compared to non-neoplastic counterparts. Cox regression analysis confirmed TMT1A as an independent prognostic factor for LUAD. Phenotypically, functional assays demonstrated that *TMT1A* expression inhibited LUAD cell proliferation and migration. Furthermore, single cell transcriptome sequencing analysis showed that TMT1A expression was positively correlated with the immune cells, especially macrophages. Mechanistically, high *TMT1A* expression was found to inhibit M2 macrophage polarization and downregulate PD-L1 expression in LUAD cells. In co-culture experiments involving LUAD cells and T cells, *TMT1A* knockdown suppressed T cell activation and reduced IFN-γ secretion. These findings were further validated by *in vivo* experiments, where *TMT1A* expression was found to promote CD8^+^ T cell infiltration in LUAD. These findings demonstrated tumor-suppressive functions coupled with its immunomodulatory capacity position TMT1A as a promising therapeutic target for LUAD treatment.

## Highlights

• TMT1A downregulation predicts poor prognosis in lung adenocarcinoma
• TMT1A inhibits LUAD cell proliferation, migration, and invasion
• TMT1A blocks M2 macrophage polarization and enhances CD8^+^ T cell activation

## Graphical abstract

**Figure:**
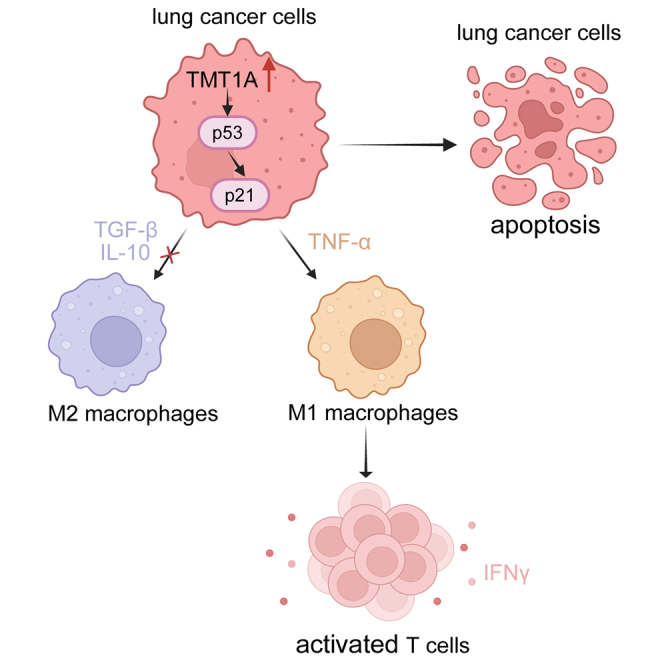

## Introduction

Lung cancer stands as the foremost cause of fatalities associated with cancer, accounting for approximately 16 million deaths annually.^1^ Among its various pathological subtypes, lung adenocarcinoma (LUAD) is the predominant form, with the majority of patients receiving a diagnosis at an advanced stage of the disease.^2^ While recent advancements in diagnostic techniques, surgical interventions, radiotherapy, chemotherapy, and immunotherapy have markedly enhanced the clinical outcomes for LUAD patients, the five-year survival rate remains disappointingly low.^3^^,^^4^ The unfavorable prognoses and the limitations inherent in current treatment modalities underscore the pressing necessity to investigate the molecular mechanisms that contribute to LUAD and to innovate more effective anti-tumor therapeutic strategies.

Tumor immune microenvironment (TME) plays an important role in tumor initiation, progression, metastasis, and therapeutic resistance of cancer.^5^ Consequently, the targeted modulation of the immune microenvironment presents a promising therapeutic strategy aimed at enhancing the clinical outcome or prognosis of patients with LUAD. Currently, immune checkpoint inhibitors, which function by inhibiting the PD-1/PD-L1 signaling pathway, have significantly augmented the treatment efficacy for lung cancer, particularly among LUAD patients exhibiting positive PD-L1 expression.^6^ Nevertheless, it is important to note that not all patients exhibit a substantial response to immunotherapy; in fact, some individuals may experience no survival advantage following treatment and could potentially suffer from severe adverse effects, which may further hasten mortality.^7^

Tumor-associated macrophages (TAMs) are key players in the tumor immune regulatory mechanism. TAMs can be polarized into two distinct phenotypes: the pro-inflammatory M1 phenotype and the immunosuppressive M2 phenotype.^8^^,^^9^^,^^10^ Growing evidence indicates that M2-polarized TAMs (M2 TAMs) exhibit tumor-promoting functions.^11^^,^^12^^,^^13^ These cells are characterized by the secretion of cytokines, including interleukin (IL)-6, IL-10, and transforming growth factor (TGF)-β, which suppress anti-tumor immune responses and ultimately drive cancer progression.^14^^,^^15^ During tumorigenesis, complex intercellular network communication is formed between malignant cells and M2 TAMs.^16^ Consequently, identifying a target to disrupt the signaling between cancer cells and M2 TAMs is crucial for impeding tumor progression.

So far, more than 170 RNA modifications have been documented, of which RNA methylation is the most common.^17^^,^^18^ These modifications exert epigenetic influences on various biological processes, including RNA stability, localization, mRNA translation, and translocation.^19^^,^^20^ Nucleoside methyltransferases, characterized by their S-adenosylmethionine-binding domain, include the methyltransferase-like (METTL) protein family that is capable of methylating mammalian RNA.^21^ TMT1A, located on chromosome 12, functions as an integrated membrane protein within the endoplasmic reticulum, facilitating the recruitment of cellular proteins to form lipid droplets.^22^^,^^23^^,^^24^ In addition, TMT1A plays a regulatory role in stem cell differentiation processes such as osteogenic and odontogenic differentiation, suggesting that it may influence the self-renewal and multi-directional differentiation capabilities of tumor stem cells.^25^^,^^26^ Alterations in *TMT1A* expression have been linked to several cancers, including liver,^27^ thyroid,^28^ breast cancers,^29^ choriocarcinoma,^30^ and LUAD.^31^ Jun et al. found that TMT1A enhances methotrexate resistance by engaging in survival-promoting signaling cascades, such as the PI3K/AKT and ERK1/2 pathways, which bolster tumor cell viability and counteract apoptotic mechanisms.^30^ Zhang et al. found that low expression of TMT1A is associated with poor clinical prognosis in melanoma, as it inhibits cancer cell proliferation, migration, and invasion via the p53 signaling pathway.^32^ Nonetheless, the role of this protein in cancer, particularly regarding its function and involvement in TME, remains inadequately explored. Currently, research into the role of TMT1A in cancer immunity is relatively scarce.

In this study, we will investigate the association of TMT1A with expression patterns and clinical prognosis in LUAD, and further deepen our insight into its mechanistic role in the TME, aiming to identify potential therapeutic targets for LUAD treatment.

## Results

### TMT1A is lowly expressed in LUAD

**Figure 1:**
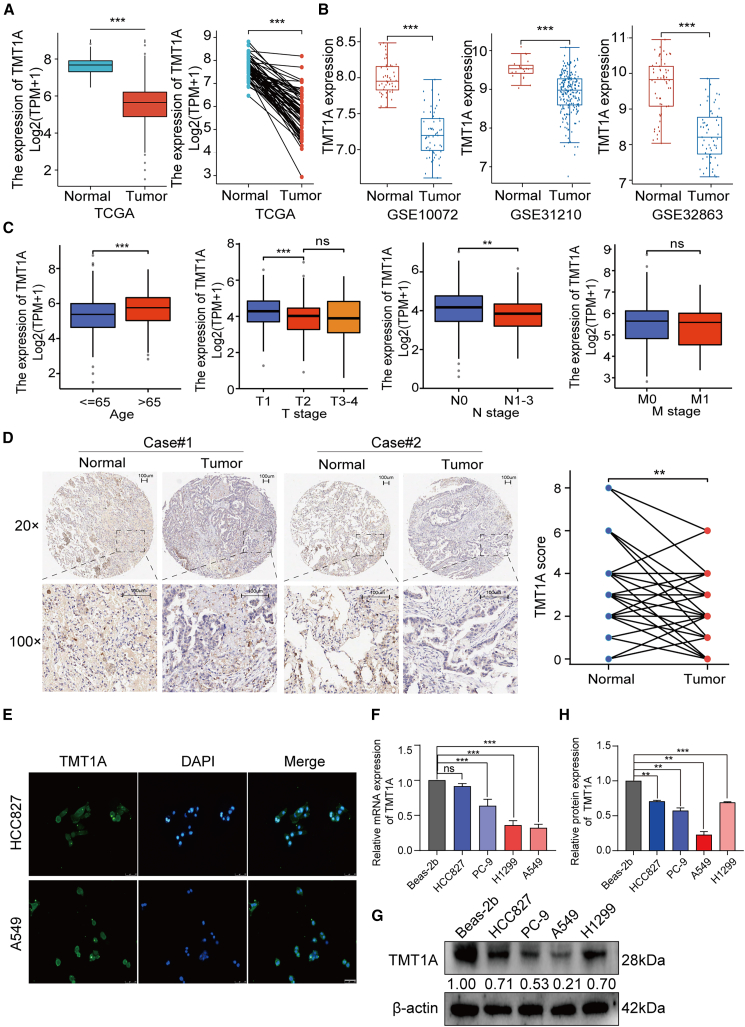
TMT1A is downregulated in lung adenocarcinoma (A) *TMT1A* expression level in unpaired (left) and paired (right) normal lung tissues and LUAD tissues based on TCGA database. (B) *TMT1A* expression level in LUAD patients based on GEO datasets. (C) Correlation between *TMT1A* mRNA expression and clinical factors in TCGA database. (D) Representative immunohistochemical (IHC) staining of TMT1A in LUAD tissues compared with adjacent noncancerous tissues. (E) TMT1A distribution in HCC827 and A549 cells was observed using immunofluorescence (IF) (scale bars, 250 μm). (F， G, and H) Representative relative *TMT1A* mRNA and protein expression levels in four LUAD cell lines relative to normal human lung epithelial cells (Beas-2b). Data are presented as mean ± SEM (A–C, F, and H). Student’s *t* test was employed to assess the two-group comparisons (A–D), and one-way ANOVA with Tukey’s test was used to analyze the multiple-group differences (C, F, and H). ^∗∗^*p* < 0.01, ^∗∗∗^*p* < 0.001. ns, no significance.

To explore the expression of TMT1A in LUAD, we used the Cancer Genome Atlas (TCGA) and Gene Expression Omnibus (GEO) datasets. In the TCGA database, *TMT1A* expression was significantly lower in LUAD tissues than that in both unpaired and paired adjacent normal tissues (Figure 1A). Consistent with the TCGA database, the Oncomine database and GEO datasets including GSE10072, GSE31210, and GSE32863 further verified the lowly expressed *TMT1A* in LUAD (Figures 1B and S1A). Next, we detected the association between *TMT1A* expression and clinical features. TMT1A was lowly expressed in patients under the age of 65 and with lymph nodes metastasis. TMT1A showed a downward trend with increasing T and M stages, although there was no significant difference between the T2, and T3-4 stages, nor M0 and M1 stage (Figure 1C). In UALCAN database, TMT1A protein expression was low in cancer tissues and further decreased with the increase in pathological stage and grade, although there was no significant difference between stages 4 and 1, which may be due to the small sample size (Figure S1B). To verify these findings, immunohistochemical (IHC) microarray was performed to detect TMT1A expression in 68 paired LUAD tissues. Compared to adjacent normal lung tissues, TMT1A protein was downregulated in LUAD tissues (Figure 1D; Table S3). Moreover, immunofluorescence (IF) analysis revealed that TMT1A was mainly distributed in the cytoplasm (Figure 1E). TMT1A was significantly downregulated in LUAD cells when compared with Beas-2b, and its expression level was the lowest in A549 cells (Figures 1F, 1G, and 1H). These results fully demonstrated that *TMT1A* is lowly expressed in LUAD.

### TMT1A expression is associated with favorable prognosis in LUAD patients

**Figure 2:**
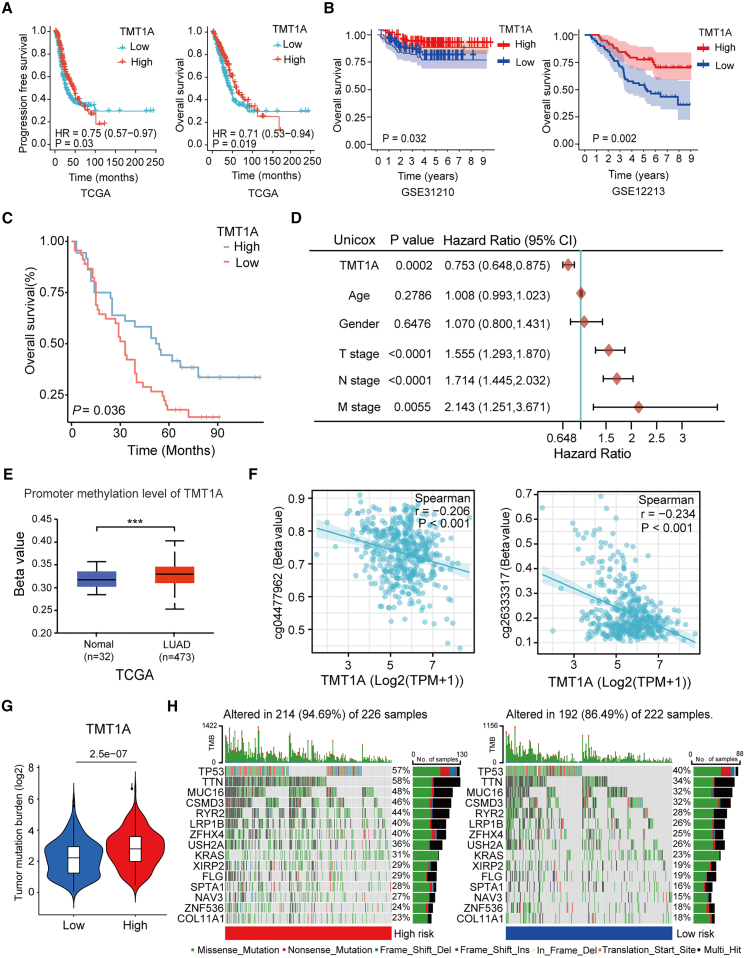
Relationship between *TMT1A* expression and prognosis of patients with LUAD (A) Kaplan-Meier curve of progression-free survival and overall survival (OS) between high- and low-TMT1A expression groups in TCGA database. (B) Kaplan-Meier curve for OS in the GSE31210 and GSE31213 datasets. (C) Kaplan-Meier curve for OS in LUAD tissue microarray. (D) Univariate Cox regression analyses were performed to determine the prognostic significance of TMT1A regarding OS in LUAD patients. (E) TMT1A promoter methylation levels in LUAD tissues and adjacent normal lung samples. (F) *TMT1A* expression was significantly downregulated by TMT1A DNA promoter CpG sites, including cg04477962 and cg26333317. (G) Tumor mutational burden (TMB) in the high- (high-risk) and low- (low-risk) TMT1A expression groups. (H) Top 15 most frequently mutated genes between the high- and low-risk of TMT1A group. Data are presented as mean ± SEM (E–G). Student’s *t* test was employed to compare the two-group (E–G), the log-rank test was used to assess the survival differences (A–C), univariate Cox regression analysis was utilized to determine the independent prognostic indicator (D) and Pearson’s correlation was performed to calculated the correlations (F). ^∗^*p* < 0.05, ^∗∗^*p* < 0.01, and ^∗∗∗^*p* < 0.001. ns, no significance.

Next, we further evaluate the relationship between TMT1A and prognosis of LUAD. Kaplan-Meier curves of LUAD using TCGA database indicated that elevated TMT1A expression was associated with longer overall survival (OS) and progression-free survival (Figure 2A). The analysis of the GEO datasets including GSE31210 and GSE12213 further confirmed that high expression of TMT1A was associated with longer OS in both early and advanced LUAD patients (Figure 2B). Similarly, the tissue microarray corresponding to clinical information was also analyzed and revealed that TMT1A expression is associated with favorable prognosis in LUAD patients (*p* = 0.036, Figure 2C; Table S4). To reveal whether TMT1A is a potential independent prognostic indicator of LUAD, we used the Cox regression analysis for TCGA dataset. The univariate analysis revealed that higher TMT1A expression was linked to improved OS (HR = 0.753; 95% CI, 0.648–0.875; *p* = 0.0002), while T, N, and M stages were significantly associated with worse OS; age and gender showed no correlation with OS (Figure 2D). The multivariate analysis confirmed TMT1A as a potential independent prognostic factor for LUAD (Figure S2A). A nomogram was constructed based on the three independent prognostic factors including TMT1A expression, T stage and N stage to predict 1-, 3-, and 5-year survival probabilities (Figure S2B) and its calibration curve showed a close match between predicted and observed outcomes (Figure S2C). These data suggested that TMT1A is a potential independent prognostic indicator of LUAD.

We further investigated the promoter methylation level of TMT1A and that was higher in LUAD tissues compared with normal tissues (Figure 2E). The methylation at CpG sites; cg04477962 and cg26333317 were significantly negatively correlated with *TMT1A* expression (Figure 2F). Tumor mutation burden (TMB) represents the gene mutation frequency in the coding region and is associated with tumor progression. Therefore, we selected 15 genes with the highest mutation frequency, such as *TP53*, *TTN*, and *MUC16*, among the LUAD samples to observe the TMB differences between the two groups. The frequency of *TMT1A* mutations in the high-expression cohort (94.69%) exceeds that observed in the low-expression cohort (84.69%) (Figures 2G and 2H). Therefore, the low expression of *TMT1A* may be caused by promoter hypermethylation and thus leading to favorable prognosis in LUAD patients.

### TMT1A expression is negative with tumor proliferation signature

**Figure 3:**
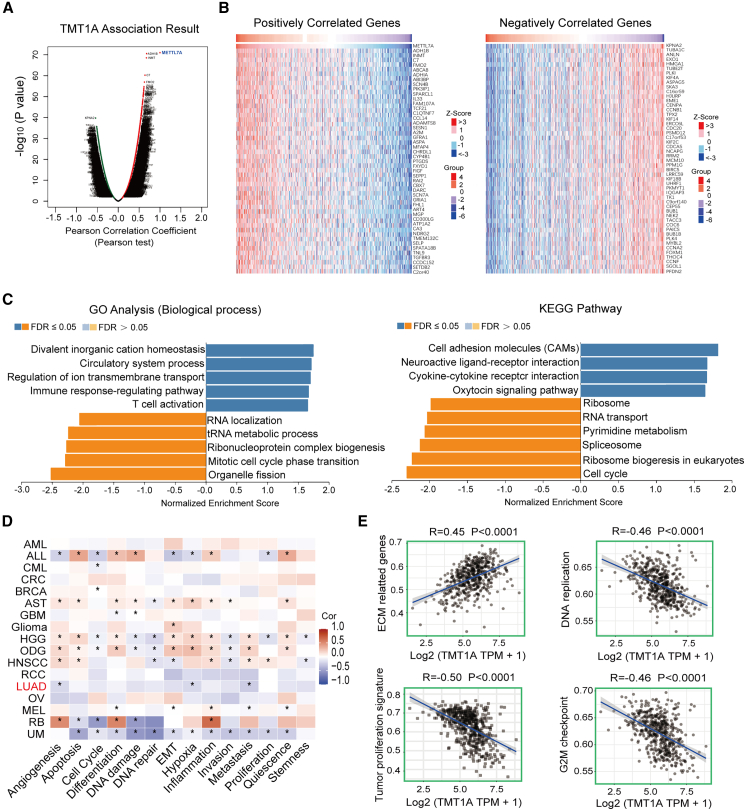
Signaling pathway regulated by TMT1A in LUAD (A) Genes highly correlated with TMT1A were assessed in the LinkedOmics database. Positive correlation with TMT1A represented by dark red dots and negative correlation indicated by dark green dots. (B) Top 50 positively and negatively co-expressed genes of TMT1A in the LUAD heatmap. (C) GO analysis highlighted significant enrichment in biological processes and KEGG pathways associated with TMT1A in LUAD. (D) CancerSEA was utilized for single-cell functional analysis of TMT1A, with red and blue indicating positive and negative correlations, respectively. (E) ssGSEA was employed to assess the correlation between TMT1A expression levels and pathway scores. Pearson’s correlation was performed to calculated the correlations (D and E). ^∗^*p* < 0.05.

To elucidate the role of TMT1A in LUAD, the LinkFinder tool on the LinkedOmics platform was utilized to analyze the co-expression network of TMT1A within the TCGA-LUAD dataset. As depicted in Figure 3A, a total of 7,496 genes exhibited positive correlation with *TMT1A* and 4,419 genes showed a negative correlation. The heatmaps show the positive and negative correlations with *TMT1A* among the top 50 genes (Figure 3B). The positive correlation was the strongest between *TMT1A* and *ADH1B* (R = 0.68), followed by *INMT* (R = 0.67). The most negatively correlated gene was *KPNA2* (R = −0.54), followed by *TUBA1C* (R = −0.51) (Figure 3B). Analysis of Gene Ontology (GO) biological processes (BP) annotations indicated that immune-related pathways including immune response-regulating signaling pathway, T cell activation, and adaptive immune response were significantly enriched in upregulated TMT1A. Cancer-related pathways, such as DNA replication and cell cycle checkpoint, were significantly enriched in downregulated TMT1A (FDR ≤0.05, *p* < 0.05) (Figures 3C and S3A). Kyoto Encyclopedia of Genes and Genomes (KEGG) pathway analysis revealed that genes co-expressed with TMT1A were predominantly associated with pathways such as cell adhesion molecules, cytokine receptor interaction, the intestinal immune network for IgA production, Th1 and Th2 cell differentiation, and Th17 cell differentiation. Negative correlations were observed in the cell cycle, DNA replication, spliceosome, and other pathways (Figures 3C and S3B).

Subsequently, we explored the single-cell functional profile of TMT1A employing the CancerSEA database. Figure 3D shows the correlation between TMT1A and the 14 functional states described in STAR Methods. These results indicate that TMT1A was negatively correlated with these pathways in LUAD. SsGSEA was used to analyze the correlation between *TMT1A* expression and pathway score. *TMT1A* expression positively correlated with extracellular matrix (ECM)-related genes (*p* < 0.0001) but negatively correlated with DNA replication, tumor proliferation signature, and the G2M checkpoint (*p* < 0.0001, Figure 3E), which were consistent with the findings from GO BP and KEGG analysis. These results indicated that TMT1A expression is negative with tumor proliferation signature.

### TMT1A overexpression inhibits the malignant phenotype of LUAD *in vivo*

For TMT1A, expression level was the lowest in A549 cells and moderately expressed in HCC827 cells (Figures 1F and 1G). We used A549 cells to construct *TMT1A* overexpression cell sublines and HCC827 cells to construct *TMT1A*-knockdown cell sublines. The knockdown efficiency of si*TMT1A*-1 and si*TMT1A*-3 was higher than that of si*TMT1A*-2 (Figures S4A and S4B); therefore, they were used in subsequent experiments (thereafter named si*TMT1A#1* and si*TMT1A*#2).

**Figure 4:**
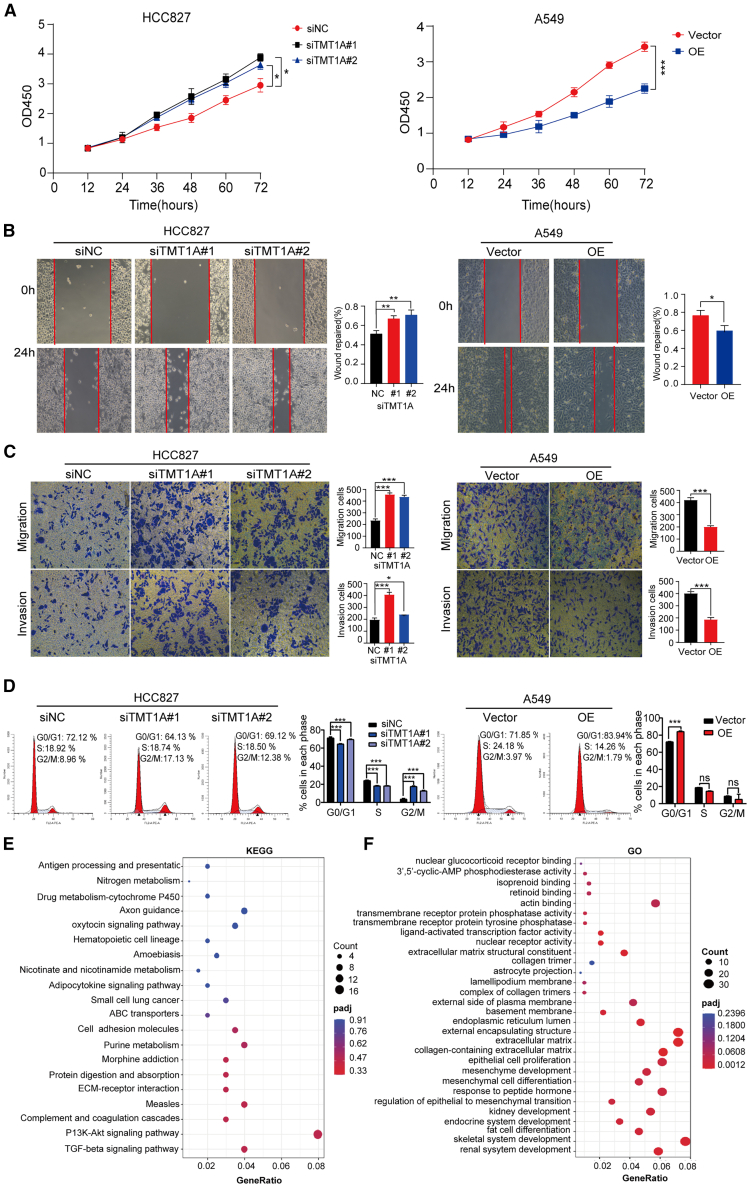
TMT1A acts as a tumor suppressor in LUAD cells (A) A cell-counting kit (CCK)-8 assay of LUAD cell proliferation under *TMT1A* overexpression or knockdown. (B and C) Cell migration was determined using wound-healing and Transwell assays (scale bars, 100 μm). (D) The effect of TMT1A on cell cycle was determined by flow cytometry. (E) GSEA plot showing enrichment of the PI3K-Akt and TGF-β signaling pathways in TMT1A-silenced cells. (F) Gene Ontology (GO) analysis of biological processes dysregulated upon *TMT1A* knockdown. Data are presented as mean ± SEM (A–D). Student’s *t* test was employed to assess the two-group comparisons (B–D) and one-way ANOVA with Tukey’s test was used to analyze the group differences (A–D). ^∗^*p* < 0.05, ^∗∗^*p* < 0.01 and ^∗∗∗^*p* < 0.001. ns, no significance.

To elucidate the biological function of TMT1A, we conducted cancer cell proliferation and migration assays. In the cell proliferation assay, *TMT1A* overexpression inhibited cell proliferation, whereas TMT1A knockdown had the opposite effect (Figure 4A).

Compared to the control group, increased *TMT1A* expression inhibited cell migration and invasion, while *TMT1A* knockdown had the opposite effect (Figures 4B and 4C). Furthermore, epithelial-mesenchymal transformation (EMT)-related protein analysis revealed that *TMT1A* overexpression led to increased E-cadherin expression and decreased Vimentin and ZEB1 expression, whereas *TMT1A* knockdown reversed these changes (Figure S4C). In addition, we further analyzed whether targeting TMT1A in LUAD cells affects the induction of cell-cycle arrest. Notably, compared to the control group, the number of cells in the G0/G1 phase increased after *TMT1A* overexpression, whereas that in the S and G2/M phases decreased (Figure 4D), suggesting that the cell cycle was arrested at the early stage of DNA synthesis. After *TMT1A* knockdown, the number of cells in the G0/G1 phase decreased, whereas that in the G2/M phase significantly increased, indicating that cells entered the DNA synthesis phase. However, the number of cells in the S phase also decreased after *TMT1A* knockdown (Figure 4D). Tumor sphere formation assays were used to evaluate the sphere-forming capacity of TMT1A. Compared to the control group, *TMT1A* knockdown in HCC827 cells markedly enhanced the sphere-forming capacity. Conversely, *TMT1A* overexpression in A549 cells markedly inhibited their tumor sphere formation ability (Figure S4D). Collectively, these findings suggest that TMT1A acts as a tumor inhibitor in LUAD.

The analysis of ssGSEA results revealed a significant positive correlation between TMT1A and the P53 pathway (R = 0.238, *p* < 0.001) (Figure S4E). As a critical tumor suppressor signaling pathway, the P53 pathway plays a pivotal role in regulating the cell cycle, facilitating DNA repair, and inducing apoptosis. To further explore this correlation, we assessed gene expression changes in the TP53 pathway. The results demonstrated that *TMT1A* overexpression significantly upregulated the expression of P53 and P21 at both the mRNA and protein levels, while *TMT1A* knockdown led to a marked decrease in their expression (Figures S4F–S4H). These findings suggest that TMT1A overexpression enhances the activity of the P53 pathway, thereby suppressing the malignant phenotype of tumors.

Furthermore, RNA sequencing (RNA-seq) analysis of TMT1A-silenced cells demonstrated the activation of oncogenic pathways, including PI3K-Akt and TGF-β signaling, and the suppression of antigen processing and presentation pathways. Gene ontology analysis further revealed dysregulation of ECM organization and EMT-related processes (Figures 4E and 4F). Collectively, these transcriptomic findings substantiate the tumor-suppressive role of TMT1A through the restraint of oncogenic signaling and the maintenance of immune homeostasis.

### TMT1A expression is essential for NSCLC immune microenvironment

**Figure 5:**
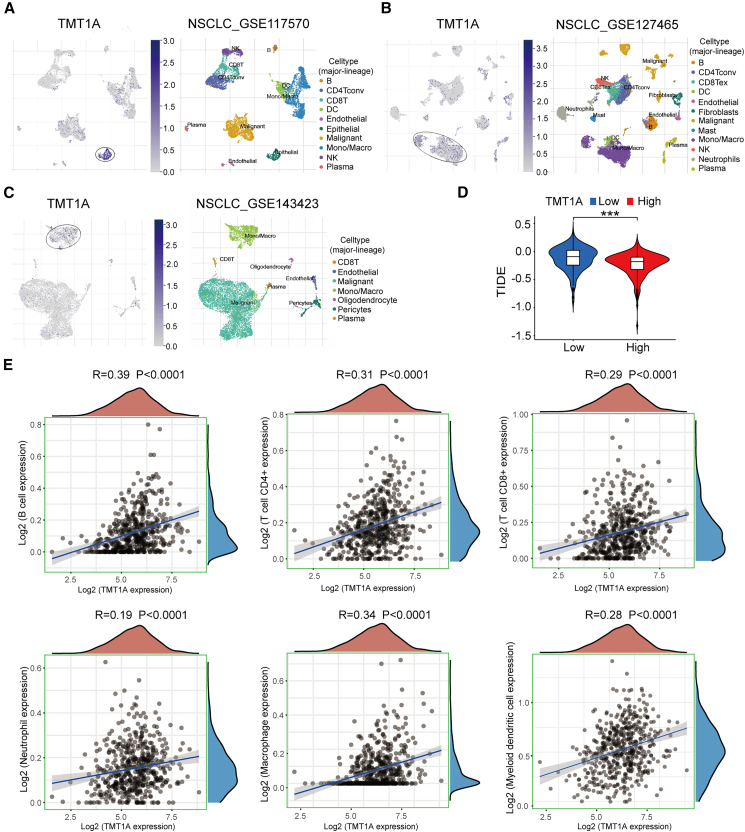
The role of *TMT1A* expression in the tumor microenvironment (A–C) Single-cell analysis of TMT1A expression. (D) Correlation between TMT1A expression and the immune cells including B, CD4^+^ T, CD8^+^ T, neutrophil, macrophage, and myeloid dendritic cells. (E) The tumor immune dysfunction and exclusion (TIDE) score between high- and low-TMT1A expression groups. Data are presented as mean ± SEM (D). Student’s *t* test was employed to assess the two-group comparisons (D), and Pearson’s correlation was performed to calculate the correlations (E). ^∗∗∗^*p* < 0.001.

Next, we used the Tumor Immune Single-Cell Hub (TISCH) database to retrieve eight non-small cell lung cancer (NSCLC) datasets (Table S4). Among them, three datasets were analyzed for all cell types (GSE117570, GSE127465, and GSE143423). Therefore, we mainly analyzed *TMT1A* expression distribution in these three datasets. Among the different datasets, TMT1A was mainly distributed in mono/macro cells, epithelial cells, and malignant cells (Figures 5A–5C). GSE143423 predominantly involved a single-cell map of multiple immunophenotypes in the NSCLC brain metastatic TME, where TMT1A was mainly distributed in mono/macro cells (Figure 5C).

The tumor immune dysfunction and exclusion (TIDE) algorithm was employed to assess the relationship between candidate genes and T cell cytotoxicity, thereby gauging resistance to immunotherapy. The TIDE score of the high-*TMT1A* expression group was lower than that of the low-expression group, indicating that this group may be more suitable for immune checkpoint blockade (ICB) therapy (Figure 5D). Functional GSEA showed that TMT1A abundance positively correlated with the immune pathway. Importantly, correlation analyses revealed that *TMT1A* expression was not only related to macrophages but also significantly associated with B cells, CD4^+^ T cells, CD8^+^ T cells, neutrophils, and myeloid dendritic cells (Figure 5E). The earlier findings suggested that *TMT1A* expression plays a key role in NSCLC immune microenvironment.

### TMT1A overexpression inhibits M2 polarization of macrophages, reduces PD-L1 in LUAD cells, and boosts T cell activation

In the earlier analysis results, the expression of TMT1A was related to immune pathways and TMT1A was mainly distributed in mono/macro, epithelial, and malignant cells in the single-cell analysis. Therefore, we intend to examine the impact of TMT1A on macrophage polarization.

**Figure 6:**
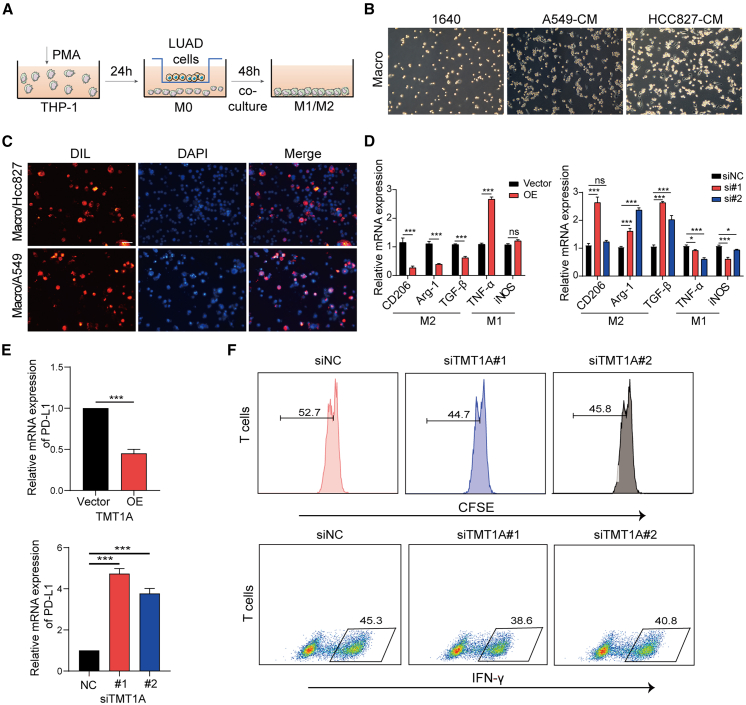
TMT1A downregulation promotes M2 polarization of tumor-associated macrophages (A) Experimental methods for macrophage generation. (B) The changes in the morphology of macrophages in the co-culture system (scale bars, 250 μm). (C) Dil staining of macrophage in a co-culture system (scale bars, 200 μm). (D) Real-time PCR analysis of mRNA expression levels of M1 and M2 markers in the co-culture system. (E) mRNA expression analysis of PD-L1 after *TMT1A* overexpression or knockdown. (F) T cell proliferation assay using CFSE staining and flow cytometry analysis of IFN-γ secretion in TMT1A knockout LUAD cells. Data are presented as mean ± SEM (D and E). Student’s *t* test was employed to assess the two-group comparisons (D and E) and one-way ANOVA with Tukey’s test was used to analyze the multiple-group differences (D and E). ^∗^*p* < 0.05 and ^∗∗∗^*p* < 0.001. ns, no significance.

THP-1 monocytes were induced to differentiate into M0 macrophages by a 24-h exposure to phorbol 12-myristate 13-acetate (PMA), followed by a 48-h co-culture with LUAD cell lines including HCC827 and A549 cells (Figure 6A). As showed in Figure 6B, the morphology of macrophages was significantly changed, and the invasive branches were significantly prolonged after the co-culture of M0 cells with LUAD cells. After LUAD cells were stained with Dil membrane stain and co-cultured with macrophages for 48 h, macrophages cells were also stained red (Figure 6C), indicating that LUAD cells secrete cellular components and influence macrophages polarization.

Furthermore, quantitative reverse-transcription PCR (RT-qPCR) results showed that the expression levels of M2 markers CD206, Arg-1, and TGF-β in macrophages increased after *TMT1A* knockout in LUAD cells, whereas the opposite trend was observed after *TMT1A* overexpression (Figure 6D). To directly assess the impact of TMT1A on the secretome of LUAD cells, we collected conditioned media from *TMT1A*-overexpressing and knockdown LUAD cells and performed ELISA. TMT1A overexpression significantly reduced the secretion of TGF-β and IL-10, whereas TMT1A knockdown markedly enhanced their production. Conversely, the level of the pro-inflammatory cytokine tumor necrosis factor alpha (TNF-α) was elevated in the supernatant of TMT1A-overexpressing cells and diminished upon *TMT1A* knockdown (Figures S5A and S5B).

Additionally, PD-L1 mRNA expression was significantly downregulated after TMT1A overexpression, while knockdown was reversed (Figure 6E). In addition, we conducted a co-culture experiment with LUAD cells and T cells. Carboxyfluorescein succinimidyl ester (CFSE) staining revealed that knockdown of TMT1A significantly inhibited T cell proliferation (Figures 6F and S5C). Flow cytometry analysis showed a decrease in interferon (IFN-γ) secretion in the *TMT1A* knockout group (Figures 6F and S5D). These results indicate that knockdown of TMT1A promotes M2-like TAM polarization and suppresses T cell activation in LUAD cells *in vitro*.

### TMT1A overexpression suppresses LUAD tumor growth and modulates TME *in vivo*

**Figure 7:**
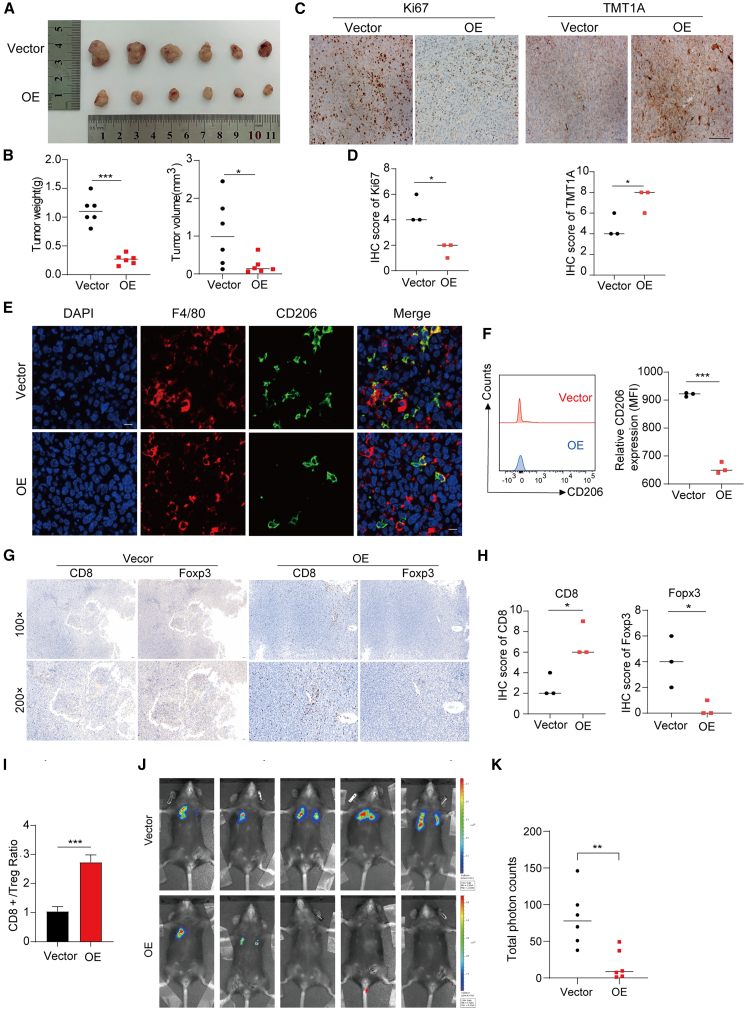
*TMT1A* overexpression inhibits LUAD tumorigenesis and modulates the TME *in vivo* (A) The subcutaneous transplanted tumor model was constructed in C57BL/6 mice (*n* = 6). (B) Xenograft volume (cm) and weight (g) were measured. (C) Representative IHC staining images showing Ki67 and TMT1A expression in the vector and OE groups (scale bars, 200 μm). (D) Ki67 and TMT1A IHC scores were quantified to compare expression between the vector and OE groups. (E and F) Representative IF images (left) or flow cytometry plots (right) showing F4/80^+^CD206^+^ M2-like macrophages within total F4/80^+^ macrophages (scale bars, 250 μm). (G and H) IHC analysis demonstrated increased CD8^+^ T cell infiltration and decreased FOPX3^+^ Tregs in TMT1A-overexpressing tumors. (I) Flow cytometry analysis of CD8^+^ T cell to Treg ratio. (J) Representative *in vivo* bioluminescence imaging of mice after tail vein injection showing lung metastatic burden. (K) Quantification of fluorescence intensity based on regions of interest (ROIs) analysis. Data are presented as mean ± SEM (D). Data are presented as mean ± SEM (B, D, F, H, I, and K). Student’s *t* test was employed to assess the two-group comparisons (B, D, F, H, I, and K). ^∗^*p* < 0.05, ^∗∗^*p* < 0.01, and ^∗∗∗^*p* < 0.001. ns, no significance.

To assess the impact of *TMT1A* expression on cell proliferation and the TME *in vivo*, we established a subcutaneous tumor model in C57BL/6 mice. As shown in Figures 7A and 7B, overexpression of *TMT1A* significantly reduced both tumor volume and weight. IHC analysis revealed a notable downregulation of Ki-67 expression in tumors overexpressing TMT1A (Figures 7C and 7D). Furthermore, IF and flow cytometry analyses demonstrated a reduction in the proportion of F4/80^+^CD206^+^ alternatively activated (M2-like) macrophages within the total macrophage population in TMT1A-overexpressing tumors (Figures 7E and 7F). Additionally, IHC analysis confirmed that TMT1A overexpression increased CD8^+^ T cell infiltration and decreased FOPX3^+^ Treg expression (Figures 7G and 7H). Flow cytometry also revealed a significant increase in the CD8^+^/Treg ratio in TMT1A-overexpressing tumors (Figures 7I and S6A). These findings suggest that *TMT1A* overexpression inhibits LUAD tumorigenesis, reduces M2 macrophage polarization, and enhances CD8^+^ T cell infiltration *in vivo*, potentially exerting therapeutic effects by modulating both macrophage activity and T cell responses.

In addition, to evaluate the metastatic potential of TMT1A *in vivo*, we established lung metastasis model. Consistent with the findings of subcutaneous transplanted tumor model, TMT1A overexpression significantly reduced the number of metastatic lung nodules compared with the vector group (Figures 7J, 7K, and S6B), further supporting its inhibitory role in LUAD progression.

## Discussion

In the current investigation, we examined the clinical attributes of TMT1A through a multi-omics approach and substantiated its functional role *in vitro* and *vivo*. Our findings revealed that TMT1A exhibits significantly downregulated expression in LUAD and functionally suppresses malignant phenotypes through multiple mechanisms: inhibiting tumor cell proliferation, impeding cancer cell migration, attenuating M2 polarization of TAMs, while simultaneously enhancing T cell activation. The demonstrated tumor-suppressive functions coupled with its immunomodulatory capacity position TMT1A as a promising therapeutic target for LUAD treatment.

Currently, TMT1A is predominantly regarded as a tumor suppressor in oncological research. In the context of thyroid cancer, a downregulation of TMT1A mRNA expression has been observed, which correlates with gene body methylation at the +4919 CpG site.^28^ In hepatocellular carcinoma, TMT1A acts as a repressor, and the adenosine-to-inosine RNA editing machinery interacts with Dicer to enhance the transition from pre-mir-27a to mature mir-27a.^27^ Consequently, mir-27a targets the 3′-untranslated region of TMT1A, leading to diminished *TMT1A* expression. A significant downregulation of *TMT1A* is noted in human mammary cells, while the murine counterpart of TMT1A is nearly absent in tumorigenic murine mammary cells.^33^ *TMT1A* expression inhibits colony formation, migration, and ECM invasion, suggesting that TMT1A may act as a tumor-suppressor gene. In our study, we observed that TMT1A was expressed at reduced levels in LUAD samples, with higher *TMT1A* expression correlating with improved prognostic outcomes. Moreover, we validated that TMT1A suppresses the malignant phenotype of LUAD *in vitro*, reinforcing the consistency of our findings with those documented in prior research.

DNA methylation represents one of the most extensively examined forms of epigenetic modification, which is regulated by the opposing actions of DNA methyltransferases and demethylases. These enzymes are crucial for preserving the structure of heterochromatin and modulating gene expression throughout developmental processes. A plethora of research has indicated that abnormal patterns of DNA methylation are implicated in the process of tumorigenesis.^34^^,^^35^ In our investigation, the TMT1A methylation level in cancer tissues was higher than that in normal tissues and negatively correlated with *TMT1A* mRNA expression. Subsequent analysis focused on specific CpG sites within the *TMT1A* promoter region, revealing a significant link between methylation at these sites and *TMT1A* mRNA expression. Importantly, the CpG sites, cg04477962 and cg26333317 displayed a negative correlation with TMT1A expression, indicating that methylation of TMT1A may exert a repressive effect on its expression.

Immunotherapy represents a significant therapeutic strategy for LUAD, with TMB emerging as an established biomarker for predicting immune responses.^36^ TMB has been identified as a potentially critical indicator of the efficacy of immunotherapy, with its correlation to response rates observed in various cancer types undergoing anti-PD-1 or anti-PD-L1 treatment. Elevated TMB levels are associated with improved responses to ICB across a diverse array of malignancies.^37^^,^^38^ In the current study, a higher frequency of gene mutations was noted in the group exhibiting elevated TMT1A expression (94.69%). Furthermore, a combined analysis of *TMT1A* expression and TMB levels revealed that patients with high TMB in the high-risk cohort experienced superior OS compared to their low-TMB counterparts. Additionally, patients with high *TMT1A* expression and high TMB had better OS rates than those with low *TMT1A* expression and low TMB. These findings align with observations in numerous malignancies, where increased TMB facilitates local immune recognition and correlates with better prognostic outcomes.

TAMs represent a significant population of stromal cells within the diverse TME, primarily derived from circulating monocytes.^39^ M2-polarized TAMs promote angiogenesis, EMT, and distant metastasis.^40^^,^^41^ In the current investigation, high *TMT1A* expression was found to inhibit M2 macrophage polarization and downregulate PD-L1 expression in LUAD cells. In co-culture experiments involving LUAD cells and T cells, *TMT1A* knockdown suppressed T cells activation and reduced IFN-γ secretion, indicating *TMT1A* expression has the potential ability to enhance immune responses and inhibit oncogenic processes, ultimately contributing to improved patient outcomes.

Previous studies have reported that *TMT1A* is downregulated in multiple cancers, including melanoma^32^ and renal cancer,^42^ where its low expression correlates with poor prognosis, reduced immune infiltration, and inferior responses to immunotherapy. Although these findings suggest that TMT1A may function as both a tumor suppressor and an immune regulatory factor, most studies to date remain limited to bioinformatic analyses. Our study provides experimental validation that addresses this gap and underscores the clinical translational potential of TMT1A. Notably, TMT1A may not only serve as a predictive biomarker for immunotherapy response, but also represent a promising therapeutic target, warranting further exploration of strategies to restore or augment its activity.

### Limitations of the study

Although our findings provide important insights into TMT1A’s dual functions, several limitations warrant consideration. First, the specific tumor-associated signaling pathways mediated by TMT1A remain to be fully elucidated. Second, the precise immunomodulatory mechanisms underlying TMT1A’s regulation of the TME necessitate systematic exploration through multi-dimensional approaches.

In conclusion, this study revealed that TMT1A exhibits significantly downregulated expression in LUAD and functionally suppresses malignant phenotypes through the regulation of the P53 pathway. Moreover, *TMT1A* expression attenuated M2 polarization of TAMs and simultaneously enhancing T cell activation. These findings position TMT1A as a promising therapeutic target for LUAD treatment.

## Resource availability

### Lead contact

Inquiries and requests for materials should be addressed to the lead contact, Yiquan Xu (xuyiquan1018@fjmu.edu.cn).

### Materials availability

All experimental reagents were either commercially available or provided by collaborators under appropriate agreements, with all sources properly documented.

### Data and code availability

• The raw sequence data from RNA-seq reported in this paper have been deposited in the National Center for Biotechnology Information (NCBI) under the BioProject no. PRJNA1359345 (https://www.ncbi.nlm.nih.gov/bioproject/PRJNA1359345).
• This paper does not report original code.
• All data needed to reanalyze the findings of this study are available from the lead contact upon request.

## Acknowledgments

This research was supported by the 10.13039/501100003392Natural Science Foundation of Fujian Province (grant no. 2025J01217); High-Level Talent Development Program of Fujian Cancer Hospital (grant no. 2023YNG14); Fujian Cancer Hospital “Outstanding Youth” Project (to Y.Q.X.); Joint Funds for the Innovation of Science and Technology, Fujian Province (grant no. 2020Y9036 to X.H.C.); and the Fujian Research and Training grants for young and middle-aged leaders in healthcare (to X.H.C.). We are grateful to the TCGA and GEO working groups for generously sharing their data. We are also grateful to Fujian Provincial Hospital Central Laboratory, Fuzhou, China, for providing the mechanics and equipment. We also thank the technical support from Wuhan Servicebio Technology Co. Ltd. (Wuhan, China) and (Shanghai, China).

## Author contributions

Y.Q.X., J.Z., and X.H.C. participated in designing this study. J.F.P. and Q.W.W. downloaded the data form corresponding databases. J.F.P., Y.N.Z., and Q.H.P. performed the bioinformatics analysis. L.H. completed the immunohistochemical staining. J.F.P., S.X.W., and Y.Q.X. participated in writing the manuscript. All the authors participated in revising the manuscript. All authors read and approved the final manuscript.

## Declaration of interests

The authors declare no competing interests.

## STAR★Methods

### Key resources table

**Table:** 

REAGENT or RESOURCE	SOURCE	IDENTIFIER
**Antibodies**		

Anti-METTL7A antibody	Abcam	Cat# ab79207; RRID: AB_10410985
Anti-METTL7A antibody	Invitrogen	Cat# PA5-96971; RRID: AB_2808773
Anti-β-actin antibody	Immunoway	Cat# YM8343; RRID: AB_3675707
Anti- Ki-67 antibody	Cell Signaling Technology	Cat#12202T; RRID: AB_2620142
Anti-F4/80 antibody	ThermoFisher	Cat# 41-4801-80; RRID: AB_2573610
Anti-CD206 antibody	ThermoFisher	Cat# 17-2061-80; RRID: AB_2637419
Anti-CD206 antibody	ThermoFisher	Cat# 17-2061-82; RRID: AB_2637420
Anti-CD8 alpha antibody	Abcam	Cat# ab217344; RRID: AB_2890649
Anti-CD8 antibody	ThermoFisher	Cat# 45-0081-80; RRID: AB_906236
Anti-Foxp3 antibody	Cell Signaling Technology	Cat#12653T; RRID: AB_2797979
Anti-CD4 antibody	ThermoFisher	Cat#12-0041-82; RRID: AB_465506
Anti-CD3 antibody	ThermoFisher	Cat# 11-0030-82; RRID: AB_464878
Anti-ZEB1 antibody	Immunoway	Cat# YM8138; RRID: Under application
Anti-E-cadherin antibody	Immunoway	Cat# YT1454; RRID: AB_3073633
Anti-Vimentin antibody	Immunoway	Cat# YM8324; RRID: AB_3073633
Anti-TP53 antibody	Immunoway	Cat# YM8533; RRID: Under application
Anti-TP21 antibody	Immunoway	Cat#YM8148; RRID: Under application
Anti-IFN gamma antibody	ThermoFisher	Cat# 12-7311-82; RRID: AB_466193

**Chemicals, peptides, and recombinant proteins**		

SPARKeasy RNA Extraction Kit	SparkJade,Shandong,China	AC0202
PrimeScript RT Master Mix	TaKaRa	RR036A
TB Green® Premix Ex Taq™ II	TaKaRa	RR820A
CFSE Cell Proliferation Kit	ThermoFisher	C34570
D-Luciferin potassium salt	Beyotime,Shanghai,China	ST196-2g
LIVE/DEAD™ Fixable NIR Kit	ThermoFisher	L34976
Foxp3/Transcription Factor Buffer	ThermoFisher	00-5521-00

**Critical commercial assays**		

Cell Counting Kit-8	Meilunbio,Dalian,China	MA0218
Apoptosis Detection Kit	Meilunbio	MA0220
Human TNF-α ELISA Kit	Reedbiotech,Wuhan,China	RE1060H
Human IL-10 ELISA Kit	Reedbiotech	RE3187H
TGF-β1 ELISA Kit	Reedbiotech	RE10013

**Deposited data**		

Raw sequence data from RNA-seq	This paper	https://www.ncbi.nlm.nih.gov/bioproject/PRJNA1359345 (BioProject: PRJNA1359345)

**Experimental models: Cell lines**		

Beas-2b	iCell Bioscience Inc.	iCell-h023
A549	iCell Bioscience Inc.	iCell-h011
H1299	iCell Bioscience Inc.	iCell-h153
PC9	iCell Bioscience Inc.	iCell-h263
HCC827	iCell Bioscience Inc.	iCell-h068

**Experimental models: Organisms/strains**		

C57BL/6 mice	Fujian Medical University	N/A

**Oligonucleotides**		

See Tables S1 and S2	This paper	N/A

**Recombinant DNA**		

TMT1A overexpression	genechem	N/A

**Software and algorithms**		

GraphPad Prism	GraphPad Software	https://www.graphpad.com
ImageJ	ImageJ	https://imagej.nih.gov/ij
GSEA software	Broad Institute	https://www.gsea-msigdb.org/gsea/index.jsp
R software	R software	https://www.r-project.org
Flow Jo software	Flow Jo, LLC	https://www.flowjo.com
Quantity One software	Bio-Rad Laboratories, Inc.	https://www.bio-rad.com

### Experimental model and study participant details

#### Cell lines and cell culture

The human bronchial epithelial cell lines Beas-2b and the A549, H1299, PC9, and HCC827 LUAD cell lines were obtained from iCell (Shanghai, China). All cell lines used in this study were tested for authenticity by short tandem repeat profiling and verified to be free of mycoplasma contamination using mycoplasma detection kit. These cell lines were cultured in RPMI 1640 medium (Meilunbio, Dalian, China), which was enriched with 10% fetal bovine serum (FBS; Meilunbio) and supplemented with antibiotics at a concentration of 1% penicillin/streptomycin (Meilunbio). Additionally, mouse Lewis lung cancer (LLC) cells were cultured in DMEM medium supplemented with 10% FBS.

#### Mouse models

##### Mouse xenograft study

In the xenograft tumor model, male C57BL/6 mice, aged 4-6 weeks, were sourced from GemPhamatech Co., Ltd., Changzhou Branch (Jiangsu, China) and housed under SPF conditions. The mice were randomly divided into two groups (n = 6) and subcutaneously injected with LLC cells (5×10^6^/100μl) stably transfected with either a *TMT1A* overexpression plasmid or an empty vector. Tumor size and weight were assessed every three daysto evaluate tumor growth, employing the formula for tumor volume calculation: 0.5 × length × width^2^. The C57BL/6 mice were euthanized on day 20 post-injection. All tumors were surgically removed and weighed. After resection in 4% paraformaldehyde, all tumors were embedded and sectioned for IHC staining. The animal experimentation protocol utilized in this investigation received approval from the Animal Ethics Committee at Fujian Medical University (IACUC-FJMU-20230155).

##### Pulmonary metastatic tumor model

To establish the lung metastasis model, male C57BL/6 mice, aged 4-6 weeks, were sourced from GemPhamatech Co., Ltd., Changzhou Branch (Jiangsu, China) and housed under SPF conditions. The mice were randomly divided into two groups (n = 6) and injected via the tail vein with 1 × 10^6^ LLC-luc cells in 100 μL PBS. The groups were as follows: 1) Vector control (LLC cells transfected with empty vector); 2) TMT1A OE (LLC cells overexpressing TMT1A). Body weight was measured every three days. Two weeks post-injection, lung tumor growth was monitored using an *in vivo* imaging system (IVIS). Prior to imaging, D-luciferin (150 mg/kg, Beyotime) was administered intraperitoneally before image acquisition. Immediately after imaging, all mice were euthanized, and lung tissues were harvested for H&E staining and subsequent analysis. The animal experimentation protocol utilized in this investigation received approval from the Animal Ethics Committee at Fujian Medical University (IACUC-FJMU-20230155).

#### Patient specimens

A human LUAD tissue microarray, designated as HLugA180Su08, was procured from the Shanghai Outdo Biotech Company. This microarray comprises 98 specimens from LUAD patients (60.20% male, 39.80% female), with 51.02% aged ≤60 years and 48.98% > 60 years. These specimens were alongside with 82 adjacent normal tissue samples. Among these, 81 samples were accompanied by comprehensive survival and clinical data. Those 81 samples were grouped according to their TMT1A expression levels, and were assigned to the corresponding experimental groups. No gender- or age-related differences in TMT1A expression were detected. The study received ethical clearance from the Clinical Research Ethics Committee of Outdo Biotech (approval number SHYJS-CP-1904014).

### Method details

#### Gene expression and prognosis analysis

The “Gene_DE” module of the “Cancer Exploration” platform, version 2 (TIMER2) web server (accessible at http://timer.cistrome.org/), was utilized with “*TMT1A*” as the search term to investigate the differential expression of the *TMT1A* gene in tumor versus normal tissues, utilizing data from The Cancer Genome Atlas (TCGA).^43^^,^^44^

Transcriptome data for LUAD, including fragments per kilobase of transcript per million mapped reads, DNA methylation profiles, and clinical data for a cohort of 535 LUAD samples alongside 59 adjacent normal tissues, were retrieved from the TCGA database (http://cancergenome.nih.gov/). Additionally, the NCBI Gene Expression Omnibus (GEO) datasets, specifically GSE10072, GSE31210, and GSE32863, were employed to assess TMT1A expression levels in cancerous tissues compared to adjacent normal tissues. For the analysis of overall survival (OS) among patients, the GSE13213 and GSE31210 datasets were utilized (http://www.ncbi.nlm.nih.gov/geo/). Furthermore, the UALCAN database facilitated the examination of correlations between TMT1A protein expression levels and various clinical parameters, including tumor stage, gender, and grade, utilizing data from the clinical proteomic tumor analysis consortium.^45^^,^^46^

#### Co-expression network and gene enrichment analysis

To investigate the gene co-expression profiles, the LinkedOmics database (http://www.linkedomics.org/login.php) was utilized.^47^ The co-expressed genes of TMT1A were visualized through a heatmap generated based on the Pearson correlation coefficient. Gene set enrichment analysis (GSEA) was employed to identify the Kyoto Encyclopedia of Genes and Genomes (KEGG) pathways and Gene Ontology (GO) biological processes (BP) that are associated with *TMT1A* and its co-expressed genes, adhering to a threshold of a minimum of three genes (FDR ≤ 0.05, *p* < 0.05). The relationship between TMT1A expression levels and pathway scores was assessed using ssGSEA. RNA sequencing data, along with corresponding clinical profiles, were sourced from TCGA database, and genes pertinent to the identified pathways were assembled and subjected to analysis utilizing the R software package “GSVA”. The “methodssGSEA” parameter was specifically employed for this analysis. Ultimately, the relationship between gene expression and pathway activity scores was quantified by calculating Spearman's rank correlation coefficients.

#### Single-cell analysis

The Tumour Immune Single-Cell Hub (TISCH) (http://tisch.comp-genomics.org/)^48^ was employed to categorize malignant, immune, stromal, and various other cell types within the tumour microenvironment. The expression levels of TMT1A in LUAD were examined across these cellular populations. CancerSEA provides single-cell maps illustrating 14 distinct functional states, which include angiogenesis, apoptosis, invasion, epithelial-mesenchymal transformation (EMT), differentiation, proliferation, DNA damage, metastasis, hypoxia, inflammation, cell cycle, DNA repair, stemness, and quiescence, encompassing a total of 25 cancer types and 41,900 individual cells [36]. This resource was utilized to comprehensively elucidate the diverse functional states associated with TMT1A in cancer cells at a single-cell resolution.

#### Analysis of immune infiltration

The correlation between TMT1A expression levels and the infiltration of six major immune cell types was analyzed utilizing the TIMER database.^43^ Additionally, the TIDE (TIDE; http://tide.dfci.harvard.edu/) algorithm was applied to estimate immune checkpoint inhibitor potential in groups stratified by high and low TMT1A expression levels, encompassing a total of 229 samples from both groups.

#### Co-culture and macrophage differentiation

THP-1 cells underwent a pretreatment with 100 ng/mL of phorbol 12-myristate 13-acetate (PMA) for a duration of 24 hours (MedChemExpress, Monmouth Junction, NJ, USA) to facilitate the differentiation into M0 macrophages. M0 macrophages were co-cultured with LUAD cells transfected with *TMT1A* plasmid (Hcc827 or A549) in a 6-well Transwell co-cultivation system (0.4 μm pore size; Corning Inc., Corning, NY, USA) to generate TAMs. Following a co-culture period of 48 hours, the macrophages were harvested to isolate TAMs for subsequent analyses.

To investigate whether LUAD cells induce macrophage polarisation by secreting cellular components, we used Cell Plasma Membrane Staining Kit with Dil (Red Fluorescence) membrane dye (Beyotime, Shanghai, China) to label LUAD cell membranes. LUAD cells, cultured in FBS-free medium, were treated with Dil dye (2.5 μL) and incubated at 37°C for 20 minutes in darkness. The supernatant was removed and cleaned with PBS three times to remove the excess Dil dye. The cells were co-cultured with M0 cells for 48 h to remove the supernatant and conduct further immunofluorescence (IF) experiments.

#### T cell proliferation and activation assay

Peripheral blood mononuclear cells (PBMCs) were extracted via density gradient centrifugation and rinsed with sterile PBS. The PBMCs were then suspended in RPMI-1640 medium containing 10% FBS and 1% penicillin-streptomycin. To stimulate T cell activation, anti-CD3/CD28 antibodies and IL-2 were added, and cells were incubated for 48 hours at 37°C with 5% CO_2_. Following this, T cells were labeled with 5 μM Carboxyfluorescein Succinimidyl Ester (CFSE) to monitor proliferation and then co-cultured with LUAD cells for an additional 48 hours. Flow cytometry was employed to analyze CFSE intensity to determine proliferation levels and to evaluate T cell activation through anti-IFN-γ staining.

#### Histopathology and Immunostaining

All tissue sections underwent a dewaxing process utilizing xylene, followed by rehydration through a series of graded ethanol solutions. Then sections were incubated with a 3% hydrogen peroxide solution solution in methanol for 10 minutes. Subsequently, the sections were incubated with primary antibodies against TMT1A (67905-1-Ig, Proteintech, China) and Ki67 (27309-1-AP, Proteintech, China) for one hour at 37°C. This step was succeeded by incubating with HRP-linked secondary antibodies for 30 minutes at the identical temperature. Haematoxylin staining was conducted at 37°C for 30 seconds. The immunohistochemical (IHC) staining results were quantified based on the percentage of positively stained cells, categorized as follows: 0 for < 5%, 1 for 5–25%, 2 for 26–50%, 3 for 51–75%, and 4 for > 75%. Additionally, the staining intensity was assessed using a scale of 0 for no staining, 1 for weak staining, 2 for moderate staining, and 3 for strong staining [37]. For IF staining, the samples were visualized using 4',6-diamidino-2-phenylindole (DAPI; #MA0128, Meilunbio) as a chromogenic agent. Tumor tissues were analyzed with the aid of Alexa Fluor 594 (F4/80) and Alexa Fluor 488 (CD206) antibodies.

#### RNA extraction and quantitative real-time PCR (qRT-PCR)

RNA was extracted using TRIzol reagent from Invitrogen (Thermo Fisher Scientific, Waltham, MA, USA), and reverse transcribed with the PrimeScript™ RT Reagent Kit (Takara Bio, Japan). Quantitative real-time PCR (qRT-PCR) was performed using SYBR Green PCR Master Mix (Takara Bio) on a StepOnePlus System (Applied Biosystems, Thermo Fisher Scientific). The assessment of gene expression fold-changes was carried out using the 2^−ΔΔCT^ method, with β-actin serving as the reference gene for normalization. The primers utilized in this investigation are detailed in Table S1.

#### Plasmids, small interfering RNA (siRNA) construction, and transfection

*TMT1A* expression plasmids were generated by inserting a coding sequence into Ubi-MCS-EGFP (GeneChem, Shanghai, China). siRNAs specifically targeting *TMT1A* were purchased from Hippobio (Chaozhou, China) and transfected into LUAD cells using Liposome transfer aid according to the manufacturer’s instructions (Meilunbio). Their sequences are shown in Table S2.

#### Cell-counting kit-8 (CCK-8) assay

Cells were plated in 96-well plates at a density of 1.5 × 10ˆ^3^ cells per well. The evaluation of cell proliferation was conducted utilizing the CCK-8 assay, following the guidelines provided by the manufacturer (Meilunbio). In summary, 10 μL of the CCK-8 reagent was introduced into the culture medium and incubated for 2 hours at 37°C within a 5% CO2 environment. Subsequently, the absorbance was recorded at 450 nm every 24 hours, totaling six measurements.

#### Western blotting

Cell lysis was performed utilizing RIPA buffer supplemented with protease inhibitors (Thermo Scientific, USA) and PMSF (Beyotime). The protein concentration was subsequently assessed via a BCA assay (Beyotime). Following this, 20-30 μg of the cell lysate underwent separation through SDS-PAGE. The proteins were then transferred onto polyvinylidene fluoride membranes (Sigma, USA) and incubated with specific primary antibodies at 4°C overnight. This was succeeded by a 1-hour incubation with secondary antibodies. The target protein bands were visualized employing an enhanced chemiluminescent reagent (Meilunbio), and the density of the protein bands was quantified using Quantity One software (Bio-Rad). The primary antibodies utilized in this procedure included β-actin (YM8343, Immunoway, China), TMT1A (Ab79207, Abcam), ZEB1(YM8138, Immunoway), E-cadherin(YT1454, Immunoway), Vimentin(YM8324, Immunoway), p53(YM8533, Immunoway), p21(YM8148, Immunoway).

#### Wound-healing and transwell assays

Briefly, for the cell wound-healing assay, the prepared cell suspensions were seeded into 6-well plates and incubated in a 5% CO_2_ atmosphere at 37°C. When the cell confluence was close to 100%, cell scratches were made using a 10 μL pipette tip. After replacing the spent medium with 1% FBS, the distance between the scratched areas at 0 h was recorded under a microscope and denoted as D0. After 48 h, the scratch area distance was measured from the same location and denoted as D48. The cell scratch migration rate was = (D0 – D48)/D0 × 100%. Cell migration was calculated using ImageJ software.

In the migration assay, around 1 × 10^4^ cells were introduced into 200 μL of serum-free medium within the upper chamber of the Transwell apparatus. For the invasion assays, the upper chamber was coated with Matrigel and subsequently incubated at 37°C for a duration of 4 hours to facilitate the solidification of the Matrigel. Subsequently, around 1 × 10^4^ cells were placed in the upper compartment, while the lower compartment was filled with 600 μL of RPMI 1640 medium enriched with 10% FBS to act as a chemoattractant. Following a 24-hour incubation, cells in the upper chamber were removed, and the lower chamber was treated with formaldehyde for fixation and then stained with crystal violet. Cell quantification was carried out using ImageJ software. The experiments were repeated at least three times to confirm the results' consistency.

#### Measurement of cytokine levels by ELISA

The levels of human TNF-α, IL-10, and TGF-β1 in cell culture supernatants were quantified using specific ELISA kits (Reed Biotech, Wuhan, China) according to the manufacturer's instructions. In brief, collected supernatants were centrifuged to remove debris. For TGF-β1 detection, samples were acid-activated prior to the assay. The procedure involved incubation of samples and standards, followed by sequential incubations with a biotinylated detection antibody and an HRP-conjugate. After the addition of TMB substrate and stop solution, the absorbance was measured at 450 nm. Concentrations were interpolated from a standard curve.

#### Flow cytometry to evaluate cell cycle

Cells underwent trypsinization and were subsequently resuspended in PBS that had been pre-chilled. Following the aspiration of the cell supernatant, 1 mL of 70% ethanol was introduced, and the cells were fixed for 24 hours at a temperature of 4°C. To each test tube, 0.5 mL of propidium iodide staining solution was added, and the cells were incubated at 37°C for a duration of 30 minutes. The distribution of the cell cycle was assessed through flow cytometry utilizing the FACScan system (Becton Dickinson, USA).

#### Tumor tissue flow cytometry analysis

Tumor tissues were dissected to remove fat, hair, and necrotic areas, then mechanically dissociated in C-tubes with DMEM containing enzyme D, R, and A for 30 minutes using the gentleMACS™ Dissociator (Miltenyi Biotec). The resulting suspension was filtered through a 70 μm filter (Fisherbrand) into a 50 mL centrifuge tube, washed with PBS, and centrifuged at 2000 rpm for 3 minutes. The cell pellet was resuspended in PBS and counted to adjust the concentration to 2.5 × 10^6^ cells/mL. For T cell analysis, antibodies against CD3, CD4, and CD8 were added, and cells were incubated at 4°C for 30 minutes in the dark. After washing, cells were fixed and permeabilized with Foxp3/Transcription Factor Fixation/Permeabilization Reagent (Catalog No. 00-5521-00, Invitrogen) for 30 minutes, followed by incubation with anti-Foxp3 antibody at 4°C for another 30 minutes. After staining, cells were washed and analyzed by flow cytometry. For macrophage analysis, antibodies against CD11b, F4/80 and CD206 were added, and the same procedure was followed. Flow cytometry was performed on a BD FACSVers™ (BD Biosciences), and data were analyzed using FlowJo software.

#### Construction and evaluation of the prognosis prediction model

Univariate and multivariate Cox regression analyses were employed to assess the potential of clinical characteristics, including age, gender, and TNM stage, as well as TMT1A expression levels, to serve as independent prognostic factors. Utilizing the “rms” and “survival” packages in R, we incorporated T and N stages along with risk scores to develop a nomogram for predicting overall survival (OS) at 1, 3, and 5 years. Subsequently, we conducted a calibration curve analysis to evaluate the clinical validity of the constructed nomogram.

### Quantification and statistical analysis

The Student’s t test was employed to assess the two-group comparisons and one-way ANOVA with Tukey’s test was used to compare the multiple-group differences. Kaplan-Meier survival analysis and log-rank test were used to evaluate the prognosis of different groups. Additionally, multi- and univariate Cox regression analysis was utilized to explore the independent prognostic indicator. Associations were assessed using Pearson’s correlation. Data are presented as the mean ± SEM from at least three independent experiments. Statistical analyses and charting were performed using GraphPad Prism (version 9) and R software (version 4.2.1). A *p* value < 0.05 was considered statistically significant for all analyses. ^∗^*p* < 0.05, ^∗∗^*p* < 0.01, and ^∗∗∗^*p* < 0.001. ns, no significance.
