# Supplementary material for: TMT1A inhibits lung adenocarcinoma progression by suppressing M2 macrophage polarization
Source: iScience. 2025 Dec 3;29(1):114332. doi: 10.1016/j.isci.2025.114332 (PMC12799785; doi:10.1016/j.isci.2025.114332)
Supplement: Document S1. Figures S1–S6 and Tables S1–S5 [file mmc1.pdf]

iScience, Volume *29*

Supplemental information

TMT1A inhibits lung adenocarcinoma progression by suppressing M2 macrophage polarization

Junfan Pan, Qiongwen Wu, Yunan Zhao, Sixuan Wu, Liu He, Qihong Pan, Xiaohui Chen, Jing Zhang, and Yiquan Xu

Figure S1

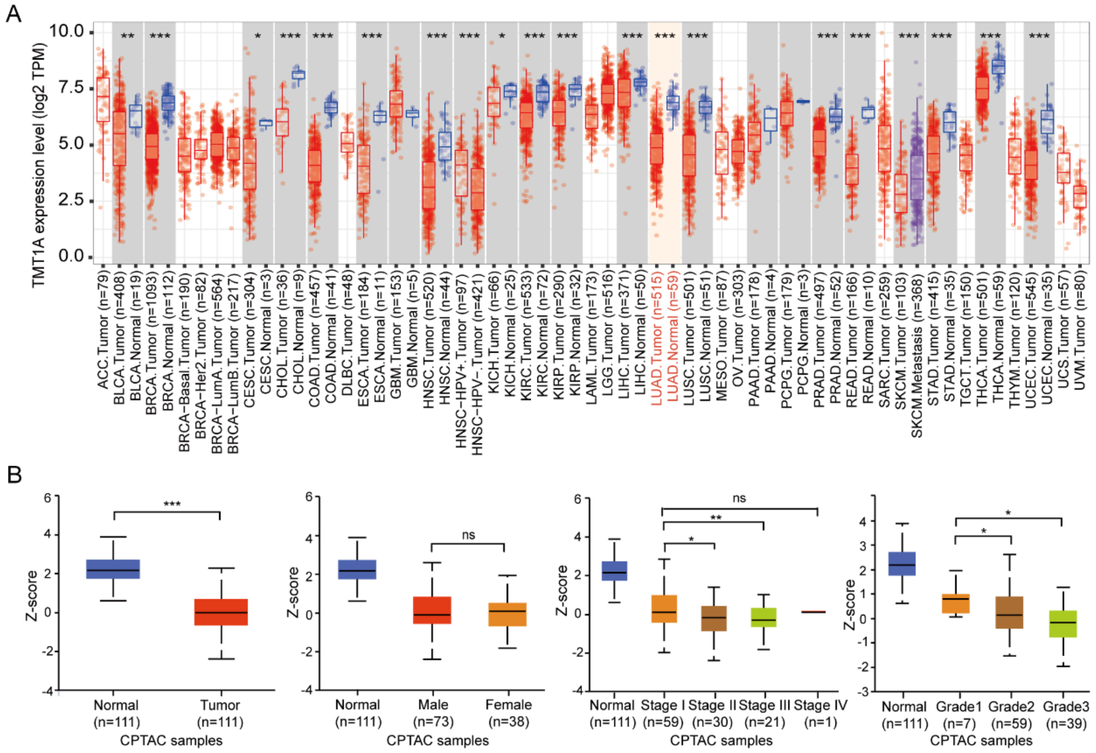

Figure S1. Analysis of *TMT1A* expression in LUAD using public database. (A) *TMT1A* was lowly expression in cancer patients, including patients with LUAD based on Oncomine database. (B) The correlation between TMT1A protein expression level and clinicopathological parameters was analysed base on the UALCAN database. Data are presented as mean ± SEM (A - B). The Student's t test was employed to assess the two-group comparisons and one-way ANOVA with Tukey's test was used to analyze the multiple-group differences (A-B). $^{*}p < 0.05$, $^{**}p < 0.01$, $^{***}p < 0.001$. ns, no significance.

Figure S2

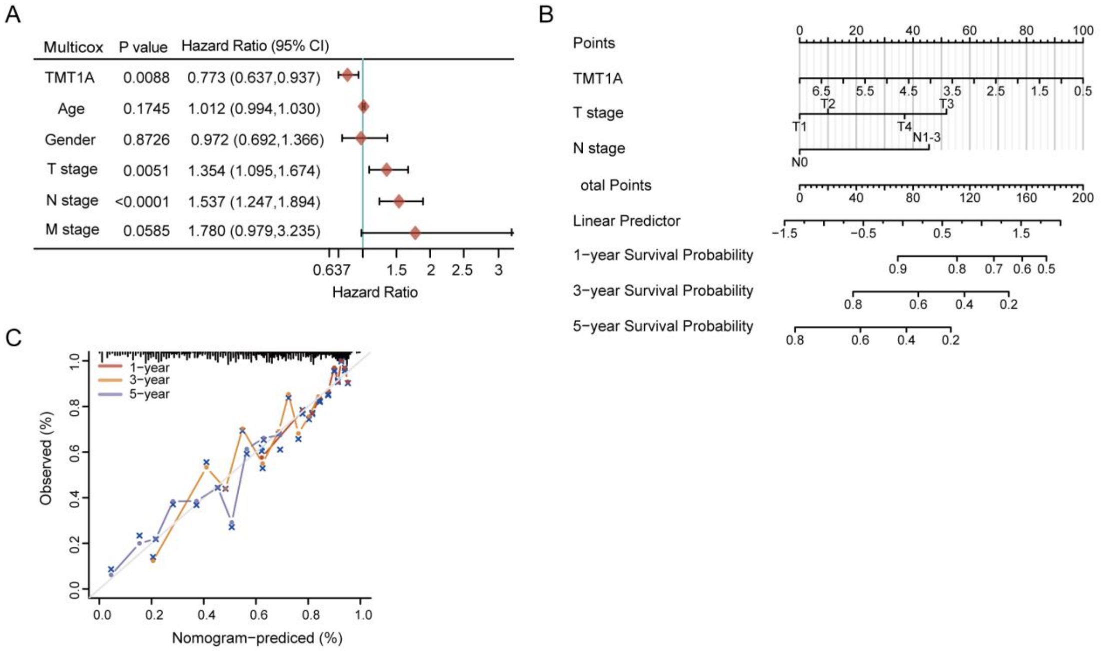

Figure S2. Identification of TMT1A as a prognostic biomarker in LUAD. (A) Multivariate Cox regression analyses were performed to determine the prognostic significance of TMT1A regarding OS in LUAD patients. (B) A nomogram was developed incorporating T stage, N stage, and TMT1A expression for prognostic prediction. (C) Calibration plots validated the concordance between the predicted and actual survival rates at 1, 3, and 5 years. The multivariate Cox regression analysis was utilized to explore the independent prognostic indicator (A). $^{*}p < 0.05$, $^{***}p < 0.001$. ns, no significance.

Figure S3

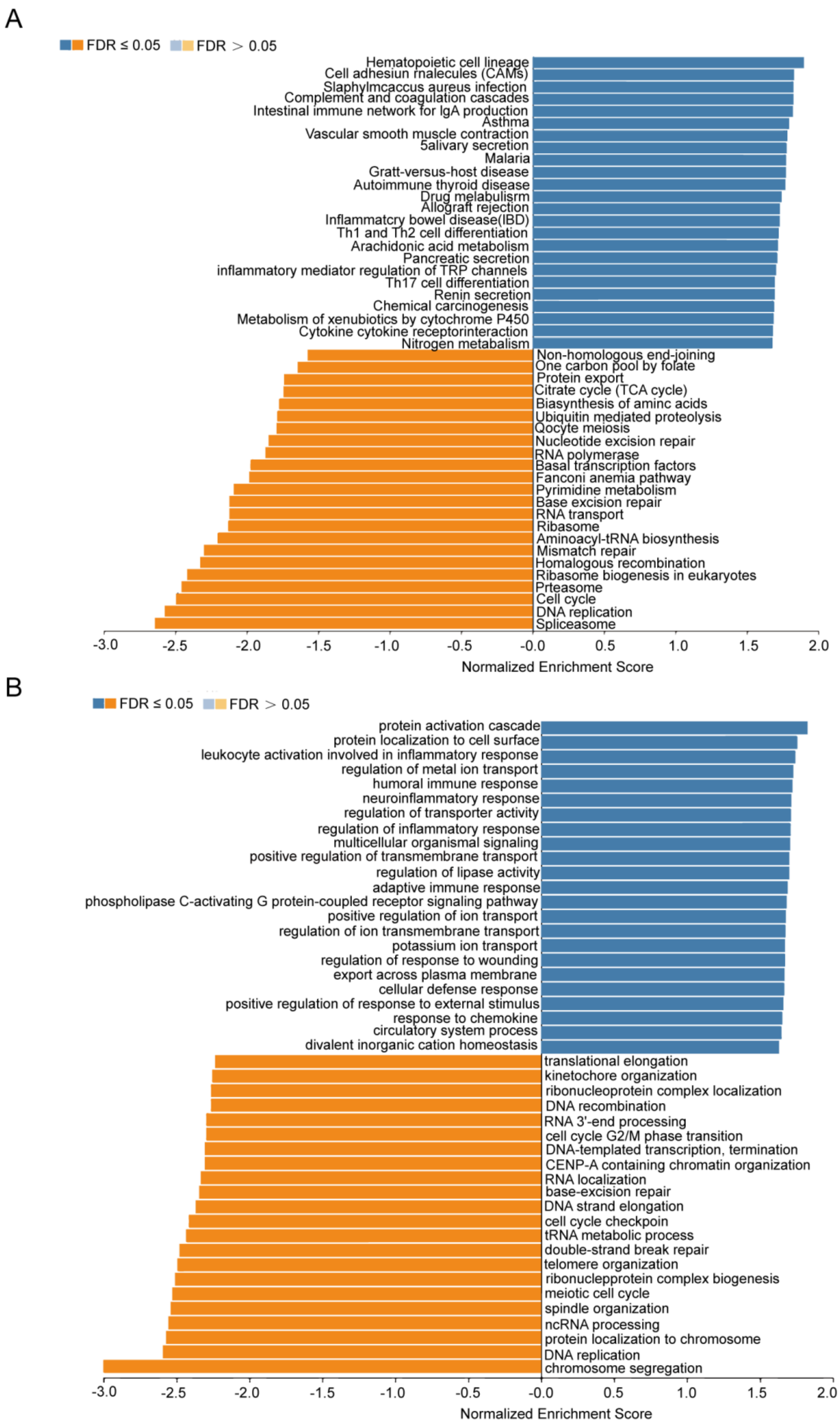

Figure S3. The enrichment pathways of TMT1A-related genes were analyzed based on LinkedOmics database.

Figure S4

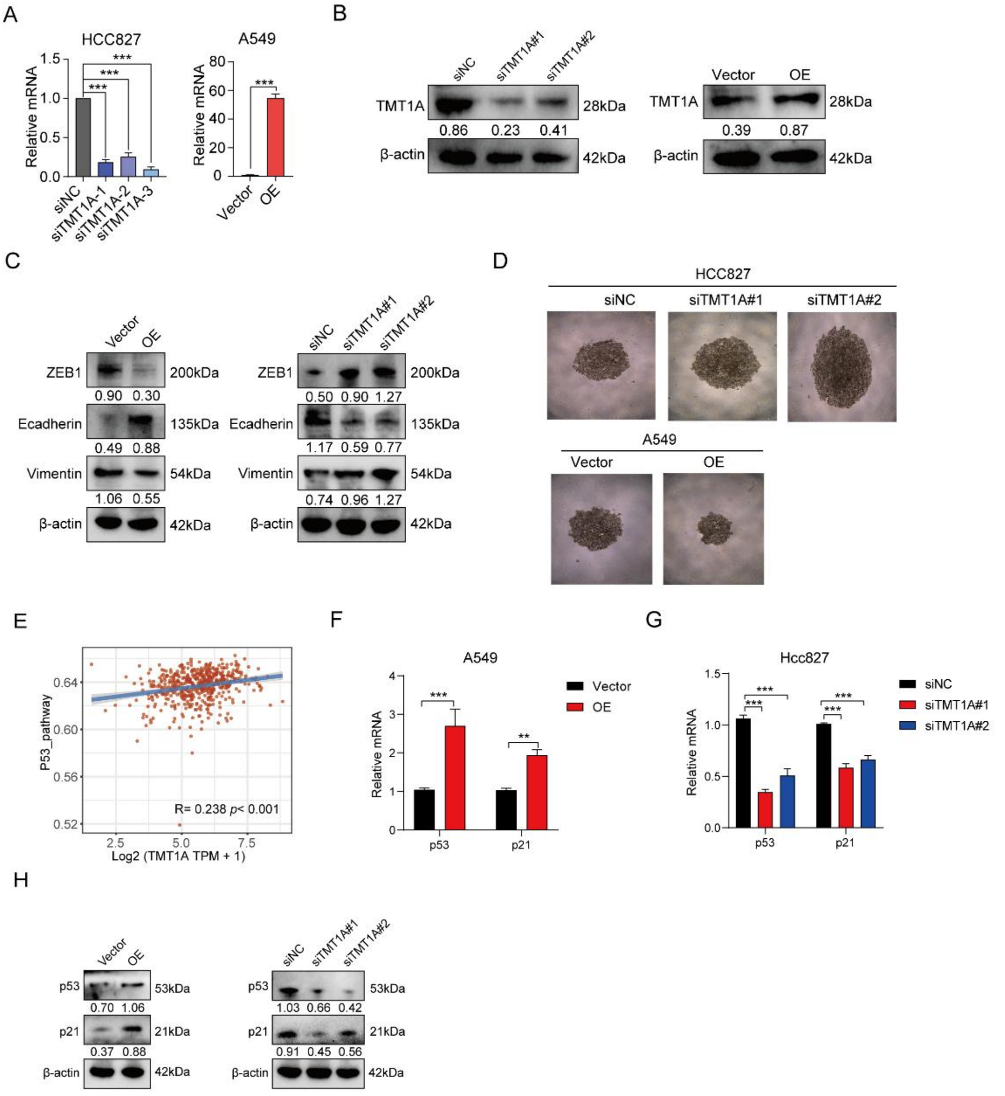

Figure S4. (A-B) Detection of knockdown and overexpression efficiency of *TMT1A*. (C) Protein levels of E-cadherin, Vimentin, and ZEB1 were detected by Western blot in the indicated cells. (D) Tumour sphere formation assays was used to evaluated the sphere-forming capacity of TMT1A (scale bars = 100 μm). (E) Correlation between *TMT1A* expression and the P53 pathway. (F)TMT1A overexpression upregulates the mRNA expression of p53 and p21 in A549 cells. (G) TMT1A knockdown downregulates the mRNA expression of p53 and p21 in HCC827 cells. (H) Protein levels of P53 and P21 were detected by Western blot in the indicated cells. Data are presented as mean ± SEM (A and F - G). The Student's t test was employed to assess the two-group comparisons (A and F), one-way ANOVA with Tukey's test was used to analyze the multiple-group differences (A and G) and Pearson's correlation was performed to calculated the correlations (E). $^{***}p < 0.001$.

Figure S5

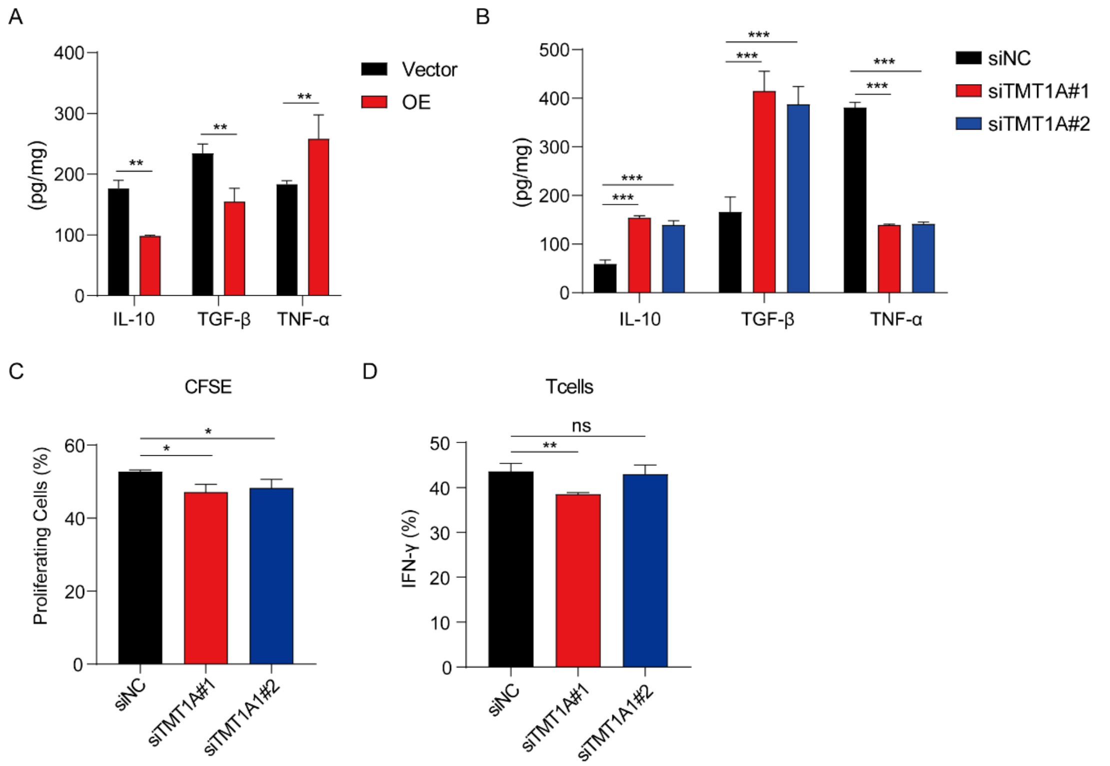

Figure S5. (A, B) Concentrations of the indicated cytokines (TGF-β, IL-10, TNF-α) in conditioned media from TMT1A-modulated LUAD cells, as determined by ELISA. (C)Statistical analysis of T cell proliferation following TMT1A knockdown. (D) Statistical analysis of IFN-γ secretion in T cells after TMT1A knockdown. Data are presented as mean ± SEM (A - D). The Student's t test was employed to assess the two-group comparisons (A), one-way ANOVA with Tukey's test was used to analyze the multiple-group differences (B - D). $^{*}p < 0.05$, $^{**}p < 0.01$, $^{***}p < 0.001$. ns, no significance.

Figure S6

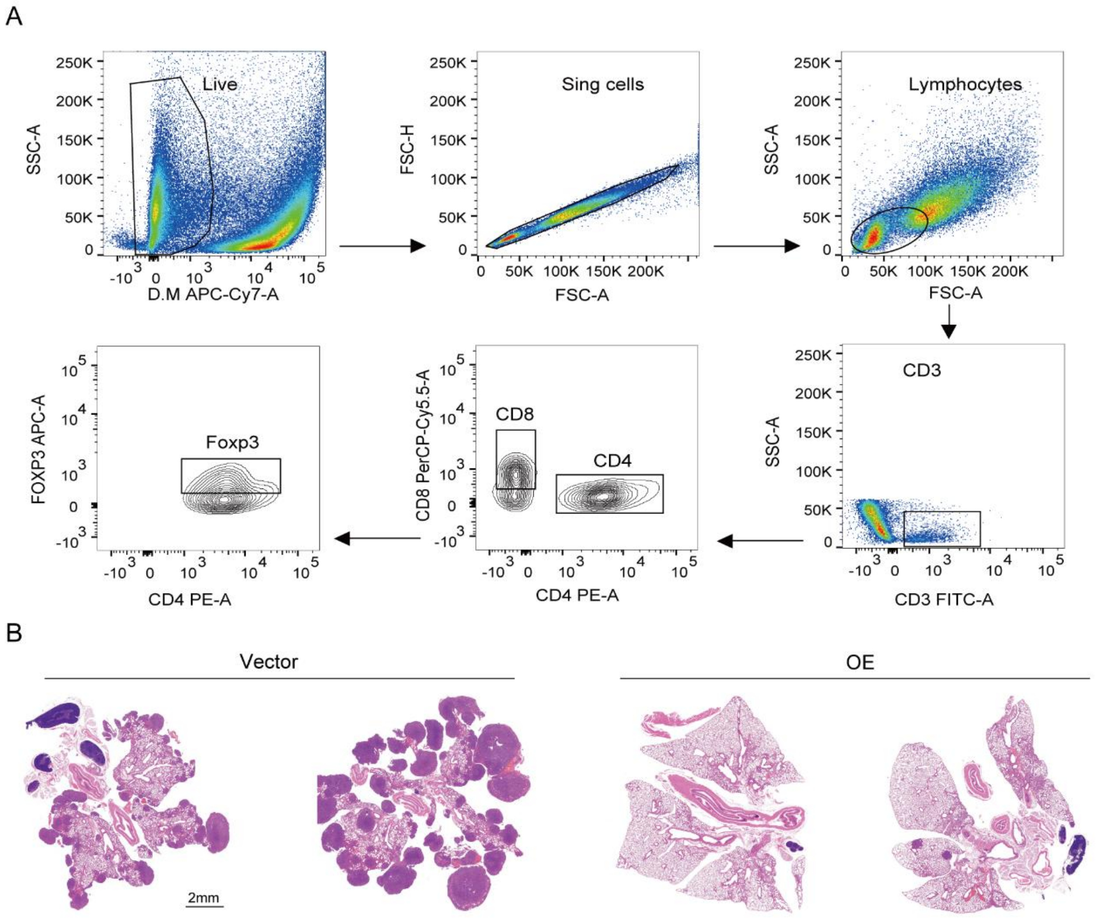

Figure S6. (A) Flow cytometry analysis showing an increased $CD8^+$/Treg ratio in tumours from the TMT1A-overexpressing group compared with the vector control. (B) Representative images of metastatic lung nodules in the pulmonary metastasis model of LUAD. $p < 0.05$, $p < 0.01$. ns, no significance.

Table S1. Primers used in this study.

Gene name		Primers
β -actin	Forward	TGACGTGGACATCCGCAAAG
	Reverse	CTGGAAGGTGGACAGCGAGG
TMT1A	Forward	CATGTGGCAGCTGAGTGTTC
	Reverse	CCAGCTCTCTCTGGTCAGGT
CD206	Forward	TGGTGAACGGAATGATTGTGTAG
	Reverse	GGTCCATCTTCCTTGTGTCAG
Arg1	Forward	GGAAGACACCAGAAGAAGTAACTC
	Reverse	GGTTAAGGTAGTCAATAGGCTTGT
TGF-β	Forward	GAGCCCTGGACACCAACTAT
	Reverse	AAGTTGGCATGGTAGCCCTT
TNF-α	Forward	TCCTCTCTGCCATCAAGAGC
	Reverse	AGTAGACCTGCCCAGACTCG
iNOS	Forward	TCACCTACTTCCTGGACATCAC
	Reverse	GAACTTCCACTTGCTGTACTCTG
PD-L1	Forward	AGGCCGAAGTCATCTGGACA
	Reverse	TGTTGATTCTCAGTGTGCTGGT
P53	Forward	CAGCACATGACGGAGGTTGT
	Reverse	TCATCCAAATACTCCACACGC
P21	Forward	CGATGGAACTTCGACTTTGTCA
	Reverse	GCACAAGGGTACAAGACAGTG

Table S2. The detailed siRNA sequences used in this study.

Gene	Sense (5'-3')	Antisense (5'-3')
hTMT1A si-1	GCUGAGUGUUCGACUUGGAAUdTdT	AUUCCAAGUCGAACACUCAGCdTdT
hTMT1A si-2	GUUGGUGCGCCCUCAUAUCUAdTdT	UAGAUAUGAGGGCGCACCAACdTdT
hTMT1A si-3	GGUUCACUGUGAUAUACAAdTdT	UUGUAUAUCACAGUGAACCdTdT

Table S3. Representative IHC staining of TMT1A in LUAD tissues compared with paired adjacent noncancerous tissues and the staining scoring analysis.

Group	Number	Min	Max	Median	Mean	SD	SE
Adjacent normal tissues	68	0	8	3	3.220	1.494	0.181
LUAD tissues	68	0	6	2	2.529	1.696	0.205

Table S4. Baseline characteristics of LUAD patients in this study.

Variables	No. of patients	Percentage (%)
**Total**	98	100
**Age (years)**		
≤60	50	51.02
>60	48	48.98
Median (range)	60（20-84）	
**Gender**		
Female	39	39.80
Male	59	60.20
**Differentiation**		
Well	7	7.14
Mod	55	56.12
Poor	36	36.73
**Pathological stage**		
I	37	37.76
II	31	31.63
III	29	29.59
IV	1	1.02
**T stage**		
T1	24	24.49
T2	51	52.04
T3	18	18.37
T4	5	5.10
**N stage**		
N0	47	47.96
N1	16	16.33
N2	15	15.31
N3	6	6.12
Unknow	14	14.29
**M stage**		
M0	98	100.00
M1	0	0.00

Table S5. Eight non-small cell lung cancer (NSCLC) single-cell datasets

Dataset Name	Species	Patients	Cells	Platform	Pri / Meta	Sorting	Publication
NSCLC_EMTAB6149	Human	5	40,218	10x Genomics	Primary	All	Lambrechts D, et al. Nat Med 2018
NSCLC_GSE117570	Human	4	11,453	10x Genomics	Primary	All	Song Q, et al. Cancer Med 2019
NSCLC_GSE127465	Human	7	31,179	inDrop	Primary	All	Zilionis R, et al. Immunity 2019
NSCLC_GSE127471	Human	1	1,108	10x Genomics	Primary	All	Newman AM, et al. Nat Biotechnol 2019
NSCLC_GSE131907	Human	44	203,298	10x Genomics	Pri, Meta	All	Kim N, et al. Nat Commun 2020
NSCLC_GSE139555	Human	6	78,829	10x Genomics	Primary	Immune	Wu TD, et al. Nature 2020
NSCLC_GSE143423	Human	3	12,193	10x Genomics	Metastatic	All	Wang L, et al.
NSCLC_GSE99254	Human	14	12,346	Smart-seq2	Primary	$CD3^{+}$ T	Guo X, et al. Nat Med 2018
